# Benefits of Some Phytochemical Compounds in Experimental Sepsis Models: A Therapeutic Perspective

**DOI:** 10.5152/eurasianjmed.2023.23382

**Published:** 2023-12-01

**Authors:** Bengul Ozdemir, Pinar Bayram, Selina Aksak Karamese

**Affiliations:** Department of Histology and Embryology, Kafkas University Faculty of Medicine, Kars, Turkey

**Keywords:** Immune system, inflammation, phytochemicals, sepsis, therapeutic effect

## Abstract

Sepsis represents a major contributor to mortality among critically ill patients, imposing a substantial economic strain on health-care systems. The uncontrolled inflammatory response during sepsis development, often leading to multiorgan failure and death, remains a challenge despite advances in antibiotics and supportive care. Factors such as antibiotic resistance, noninfectious causes, and delays in treatment initiation contribute to the complexity of managing sepsis. This review explores alternative treatment strategies focusing on the ameliorative effects of widely consumed phytochemicals and their cellular and molecular mechanisms in various in vitro and in vivo sepsis models. Preemptive administration of phytochemicals before sepsis onset appears effective, emphasizing their potential as adjunct or complementary therapeutic agents for critically ill sepsis patients. As a consequence, phytochemicals can be included in treatment regimens to enhance overall effectiveness and expedite the healing process.

**Main Points**

- Sepsis is characterized by the systemic inflammatory response to an infection and can be attributed to causes falling into 2 main categories: infectious and noninfectious factors.
- There are some different processes in the mechanism and pathophysiology of sepsis including inflammation, early activation genes, the C5a–C5a receptor axis, immune suppression, endothelial barrier dysfunction, coagulation, and clot formation.
- In experimental sepsis models, different therapeutic strategies can be used such as inhibiting secondary signaling pathways, using antimicrobial agents, administering TLR antagonists, neutralizing LPS, and inhibiting related mediators to treat sepsis or at least reduce its complications.
- Preemptive administration of phytochemicals before sepsis onset appears effective, emphasizing their potential as adjunct or complementary therapeutic agents for critically ill sepsis patients.
- As a consequence, phytochemicals can be included in treatment regimens to enhance overall effectiveness and expedite the healing process.

## Introduction

Sepsis is characterized by the systemic inflammatory response to an infection. It progresses through 3 stages of varying severity: (i) sepsis, (ii) severe sepsis, and (iii) septic shock. The significance of it lies in its occurrence in 10 out of 1000 hospitalized individuals, with 30% of these cases developing multiple organ dysfunction syndrome. Mortality rates are noteworthy, affecting 20% of sepsis patients and rising to 60%-80% in those experiencing septic shock. Timely diagnosis and intervention are imperative given the elevated risks of mortality associated with sepsis.^1,2^

Sepsis can be attributed to causes falling into 2 main categories: infectious and noninfectious factors. It may develop due to bacteria, viruses, fungi, or parasites, and it can also occur in noninfectious contexts, including systemic inflammatory response syndrome, severe trauma, surgical injuries, drug reactions, autoimmune and neoplastic diseases, pneumonia, pancreatitis, and events like urinary system infections.^3^ The frequency of microorganisms leading to sepsis varies depending on whether sepsis develops within or outside the hospital setting. the most known gram-positive and gram-negative bacteria including *Escherichia coli*, *Klebsiella* spp., *Staphylococcus* spp. are the most frequently encountered microorganisms in sepsis patients.^4^ In addition to those bacteria, viral, fungal, or parasitic infections can manifest with symptoms similar to sepsis.^2^

The main risk factors of sepsis encompass both a patient’s susceptibility to infection and the probability of acute organ dysfunction. Numerous recognized risk factors contribute to infections that frequently lead to severe sepsis and septic shock. These include chronic diseases such as acquired immunodeficiency syndrome (AIDS), Chronic obstructive pulmonary disease (COPD), and various cancers, as well as the use of immunosuppressive agents. While risk factors for infections leading to severe sepsis are well-established, those contributing to organ dysfunction are not as extensively studied. The factors influencing the occurrence of severe sepsis probably encompass the causative organism, the patient’s genetic composition, their current health status, the functioning of preexisting organs, and the promptness of therapeutic intervention. Additionally, age, gender, and racial or ethnic background contribute to the incidence of severe sepsis, with elevated rates noted in infants and the elderly, males, and the black population compared to other demographic groups.^5,6^

There are some different processes in the mechanism and pathophysiology of sepsis including inflammation, early activation genes, the C5a–C5a receptor axis, immune suppression, endothelial barrier dysfunction, coagulation, and clot formation.^7^ At its core, sepsis which is defined as an inflammatory condition triggered by the activation of the innate immune system. In sepsis, the innate immune response begins with the simultaneous recognition of microbial products related to infection and internal danger signals by various receptors such as complement, Toll-like receptors, NOD-like receptors, RIG-like receptors, mannose-binding lectin, and scavenger receptors. These receptors are mainly found on immune, epithelial, and endothelial cells strategically placed to monitor the local environment continuously. The interaction between PAMPs or DAMPs and these receptors initiates a complex intracellular signaling system with redundant and complementary activities. Furthermore, the activation of these cellular signaling pathways eventually results in the expression of some genes related with some cellular mechanisms including inflammation. Identifying the components (antigens) of bacteria, viruses, fungi, and host tissue injury products triggers the releasing of pro-inflammatory mediators, and initiating the activation of early response genes. These characteristics collectively establish a common response pattern in innate immunity, with the intensity and direction regulated by the levels of PAMPs and DAMPs and the activated signaling pathways. The overlapping yet distinct early inflammatory response to various infections and tissue injuries, including those caused by gram-negative bacteria, gram-positive bacteria, fungi, and viruses, can be attributed to the complementary nature of these pathways.^7-9^

The efficacy of sepsis treatment hinges on early diagnosis, prompt initiation of appropriate antibiotic therapy, supportive treatment, and addressing the underlying disease. Prevention stands out as a crucial avenue for reducing morbidity and mortality rates, especially given the predominantly nosocomial nature of most sepsis cases. The treatment of sepsis can be categorized into 2 main areas: appropriate antimicrobial treatment and comprehensive supportive care. Each patient in the sepsis spectrum should undergo a thorough evaluation based on available resources, and consultations should be sought as needed. Antibiotics are typically administered empirically until the active microorganism is identified. The initial 6 hours following the manifestation of sepsis symptoms are critical for prognosis. Appropriate antibiotic treatment has been shown to halve the incidence of shock in sepsis caused by gram-negative bacteria, regardless of the underlying disease.^2,10^ In current literature, there are some approaches to preventing or decreasing the efficiency of sepsis as well as known clinical treatment options. While some of these approaches have failed, studies on others are ongoing.

In experimental sepsis models, different therapeutic strategies can be used such as inhibiting secondary signaling pathways, using antimicrobial agents, administering Toll-like receptor (TLR) antagonists, neutralizing lipopolysaccharide (LPS), and inhibiting related mediators to treat sepsis or at least reduce its complications.^1^ In current literature, there are numerous studies that showed the beneficial effects of phytochemicals and their derivatives on different experimental sepsis models such as cecal ligature and puncture (CLP) sepsis model named also polymicrobial sepsis model, LPS-induced endotoxemia, and administration of viable pathogens (*Klebsiella *spp.*, E. coli* etc.). There are also some agonist/antagonists such as and other phytochemicals which are effective in sepsis models such as gossypin,^11^ luteolin,^12^ suberosin,^13^ and umbelliferone^14^. By the way, there are also new therapeutic targets rasagiline, 5-HT7 receptor agonist, salbutamol, milrinone, aliskiren, neprlysin, amiodarone, lithium, montelukast, roflumilast, alpha-lipoic acid, and sildenafil for preventing sepsis. Propofol administration has demonstrated positive effects on lung inflammation following intravenous endotoxin exposure in rat studies.^15-27^ In this review, we will review the most studied top 10 phytochemicals and their beneficial effects in different experimental models (Table 1). The reviewed phytochemicals and their beneficial effects on at least 3 sepsis models can be seen in Table 1.

## Phytochemicals on Sepsis Models

### Green Tea

Tea (*Camellia sinensis*) stands as the second most consumed beverage worldwide, trailing only water. Lately, there has been a growing curiosity in investigating the potential health advantages of tea and its contribution to preventing diseases.^28^ The literature currently encompasses over 20 000 studies investigating the relationship between green tea and its major components in the context of sepsis. Epidemiological studies have indicated that regular green tea consumption may lower the risk of cancer and heart disease. Furthermore, green tea demonstrates additional beneficial effects, including antioxidant, anti-inflammatory, antiviral, antimutagenic, antitumorigenic, antiangiogenic, and antiproliferative effects.^29^ Despite the complex composition of green tea (consisting more than 2000 components), catechins which are a group of the polyphenolic flavonoids are the most known and extensively studied. Beyond their antioxidant properties, catechins, especially epigallocatechin-3-gallate (EGCG), have demonstrated inhibitory effects on several proteins involved in inflammation, including the transcription factors nuclear factor kappa B (NF-κB) and activator protein 1. Notably, EGCG has been identified as a potent inhibitor of tumor necrosis factor alpha (TNFα)- and interleukin 1 beta (IL-1-β)-mediated NF-κB activation. Additionally, EGCG has been shown to suppress the gene expression of NOS2 in peritoneal macrophages.^30-33^

### Curcumin

Curcumin, a major component in the rhizome of turmeric, is a beneficial agent for indigenous medicine and boasts a diverse range of physiological and pharmacological activities. The literature currently encompasses nearly 20 000 studies exploring the effects of curcumin in the context of sepsis. Studies have indicated that curcumin exhibits efficacy in managing chronic inflammatory conditions, encompassing ailments such as rheumatism, atherosclerosis, type II diabetes, and cancer.^34^ Additionally, various animal models and clinical studies have demonstrated the exceptional safety of curcumin even at high doses.^35^ Current literature demonstrated that curcumin has a potential to protect from the harmful effects of LPS-induced septic shock by enhancing survival rates and alleviating organ dysfunction.^36^ Cytokine expression, NF-κB inhibition, ROS clearance and suppression of apoptosis mechanism are the underlying mechanisms of this process. Curcumin’s ability to reduce inflammation is linked to its capacity to inhibit the TLR4/NF-κB signaling pathway.^37^ It can trigger the overexpression of inflammatory mediators by inhibiting the phosphorylation of MAPKs and inhibiting the NF-κB signaling pathway activation. Additionally, curcumin restrained the production of IL-6 by LPS-induced mouse macrophage cells with nearly the same mechanism. Furthermore, curcumin inhibited M1 macrophage polarization by reducing the expression of TLR-4 and inhibiting the related signaling pathway.^38,39^

### Resveratrol

Resveratrol is one of the most known polyphenolic compound found in many plant sources such as grapes, vine, peanuts, cranberries, blueberries. As known, it is synthesized by plants undergoing infectious or ionizing radiation.^40^ The current literature includes nearly 18 000 studies exploring the effects of resveratrol in the context of sepsis. Resveratrol has been extensively studied for its diverse health-beneficial effects, including antidiabetic, antioxidant, antiaging, antiproliferative, anti-inflammatory, and cardiovascular protection.^41^ It also exhibits antimicrobial activity against a broad range of bacterial pathogens.^42-44^ Furthermore, its therapeutic effects during sepsis process and the molecular mechanisms have been thoroughly investigated in experimental sepsis models. Studies have shown that resveratrol decreases MIP-2 and IL-18 while increasing IL-10 in the bronchoalveolar lavage fluid of rats with sepsis-induced acute lung injury (ALI).^45^ In sepsis-induced liver injury, resveratrol reduces serum levels of HMGB1, decreases IL-6 and NO, and inhibits the cytoplasmic translocation of HMGB1 by suppressing the related signaling pathways including NF-κB. Upstream from HMGB1, resveratrol mediates the overexpression of sirtuin 1, downregulating HMGB1 acetylation and inhibiting its translocation to the cytoplasm, thereby ameliorating sepsis-induced liver injury.^46^ Additionally, resveratrol suppresses the overexpression of pro-inflammatory cytokines via sirt1 activation, reducing the severity of LPS-induced ALI. Resveratrol has also been reported to increase the levels of the anti-inflammatory cytokine IL-10 in acute kidney injury. Moreover, it provides a safeguard against sepsis-related encephalopathy by suppressing the NLRP3–IL-1-β axis in microglial cells stimulated with LPS.^47,48^

### Quercetin

Quercetin, found abundantly in vegetables and fruits as a widespread flavonoid, exhibits a range of biological functions. These include the regulation of oxidative stress, anti-infectious properties, anti-inflammatory effects, and activities that support neuroprotection.^49^ The current literature encompasses nearly 17 000 studies investigating the effects of quercetin in the context of sepsis. The majority of these studies suggest that quercetin can reduce the release of certain pro-inflammatory cytokines, such as TNF-alpha and IL-1, primarily by blocking the activation of MAPK and NF-κB pathways.^50^ On the other hand, quercetin has the potential to prevent the transcriptional generation of NO through the activation of macrophages and microglia.^51^ The suppression of inducible iNOS expression by quercetin is accompanied by the inactivation of NF-κB and STAT1, along with an increase in HO-1 or IL-10 expression.^49^ Considering that macrophages are one of the most important immune cells of the innate immunity and play a crucial role in the pathogenesis of sepsis, studies showed that quercetin has an ability to inhibit M1 macrophage polarization and the production of M1-related inflammatory cytokines in fibrotic livers.^52^

### Berberine

Berberine (from *Berberis vulgaris*), an isoquinoline alkaloid, is commonly found in Chinese herbal medicines.^53^ With a molecular weight of 336.37 kDa, berberine can be obtained through de novo synthesis. The current literature includes nearly 6500 studies exploring the effects of berberine in the context of sepsis. While berberine was initially approved as an antibacterial agent, it has demonstrated therapeutic potential in some diseases including cardiovascular and neurodegenerative diseases.^54^ Berberine has the capacity to interact with a variety of molecular targets, attaching to active spaces characterized by specific structural and physicochemical properties. The effectiveness of berberine about treating the sepsis have been extensively explored in cellular and animal models, although there are no clinical studies on berberine for treating sepsis in practice.^55^ In the context of sepsis-related lung injury and ARDS, berberine’s protective role primarily stems from its anti-inflammatory effects. Berberine has an anti-inflammatory effects, potentially related with inhibiting the NF-κB signaling pathway activation. This, in turn, leads to the inhibition of gene expressions and productions of proinflammatory mediators. Additionally, berberine suppresses the phosphorylation of MAPKs, along with reducing the level of reactive oxygen species in macrophages.^54,56^ Berberine pretreatment has been shown to maintain the integrity of the endothelial glycocalyx, inhibit NF-κB signaling pathway activation, and decrease the releasing of proinflammatory cytokines such as TNF-α, and IL-6 in LPS-induced ARDS mice model.^57^ Studies have also reported that berberine inhibits proinflammatory cytokine expression in bone marrow-derived macrophages and improves survival in septic mice by suppressing NF-κB- and IL-6-mediated STAT3 activation.^58^ These findings suggest that the anti-inflammatory roles may be the core mechanisms by which berberine treats sepsis-related lung injury.

### Genistein

Genistein, a major soybean phytoestrogen and receptor tyrosine kinase antagonist, exhibits diverse biological activities, including antioxidant, anti-inflammatory, and hypolipidemic actions.^59,60^ The current literature comprises nearly 6000 studies exploring the effects of genistein in the context of sepsis. Three studies investigating blood pressure and the vasopressor reaction to catecholamines after the intraperitoneal injection of genistein, given either before or concurrently with LPS, revealed significant findings.^61-63^ Lipopolysaccharide, by increasing vascular iNOS expression and promoting NO production, leads to pronounced vasodilation and vasoplegia. This occurs through the activation of cyclic guanosine monophosphate and nitrosylation of potassium [K+] channels in vascular smooth muscle cells. In 2 out of 3 studies, the drop in blood pressure induced by LPS was attenuated by genistein.^61,62^ The positive circulatory effects of genistein were duplicated with experimental tyrosine kinase inhibitors but not with daidzein, another soy flavonoid that possesses phytoestrogenic properties but lacks tyrosine kinase inhibitory activity. Treatment involving an experimental iNOS inhibitor similarly sustained adequate hemodynamics, and the safeguard provided by genistein correlated with diminished iNOS expression in both vascular and pulmonary contexts and a reduction in plasma NO metabolites. Additionally, genistein was observed to mitigate LPS-induced activation of TLR-4 tyrosine kinase, consequently hindering NF-κB-mediated transcription of iNOS.^64^

### Apigenin

Apigenin, a prevalent dietary flavonoid found in plants, demonstrates diverse biological functions, encompassing anticancer, antidiabetic, antifungal, antiviral, antibacterial, anti-inflammatory, and antioxidant properties across different cellular processes.^65^ The current literature encompasses nearly 4500 studies exploring the effects of apigenin in the context of sepsis. Several studies have reported that apigenin displays robust anti-inflammatory activity in both in vitro and in vivo models as well as inhibiting of COX-2 expression, cellular adhesion molecules, and the adhesion between monocytes and human umbilical vein endothelial cells.^66,67^ A study reported that pretreatment with a different doses of apigenin caused a significant decrease in the number of inflammatory cells in CLP-induced sepsis model.^65^ Apigenin also effects the production of pro- and anti-inflammatory cytokines and some mediators such as IL-1, IL-8, IL-6, and TNFα.^68^ It makes this effects by primarily through the inhibition of p65 phosphorylation, without influencing LPS-stimulated IKK degradation or the NF-κB-DNA binding capacity. Further investigations show that apigenin elevates the concentrations of GSK3β, Nrf2, and HO-1 as well as NF-κB expression.^69^ In the context of sepsis, extensive generation of reactive oxygen species (ROS) triggered by the activation of numerous neutrophils is the main contributor to organ tissue damage. Apigenin and its derivatives, known for their outstanding antioxidative effects, play a role in treating various diseases by effectively restraining oxidative stress.^70^ Apigenin can inhibit ROS generation and downregulate the expression of proinflammatory cytokines as mentioned above. On the other hand, apigenin’s antioxidant mechanisms encompass the inhibition of oxidase activity, modulation of redox signaling pathways, augmentation of both enzymatic and nonenzymatic antioxidants, metal chelation, and the scavenging of free radicals.^71^

### Paeoniflorin

Paeoniflorin, a water-soluble bioactive component of *Paeonia lactiflora* Pall, exhibiting significant potential for anti-inflammatory, antimicrobial, antioxidant, immunomodulatory, anticancer, and anti-ischemic properties, particularly in injury models.^72,73^ The literature encompasses nearly 4500 studies examining the effects of paeoniflorin in the context of sepsis. A comprehensive review highlighted the crucial roles of paeoniflorin in cellular processes, including the regulation of G-protein-coupled receptors, NF-κB, and MAPKs signaling, as well as the modulation of B lymphocytes, T lymphocytes, and dendritic cells.^73^ Moreover, paeoniflorin demonstrates protective effects against LPS-induced cellular toxicity in mouse macrophage cells by increasing the number of viable cells, inhibiting the production of some mediators such as NO and PGE2, and DNA damage repair.^74^ It effectively blocks the inflammatory response induced by LPS, modulates the F-actin expression and the phosphorylation of PI3K/Akt, PKC, and cofilin.^75^ In peripheral blood mononuclear cells from patients with primary Sjögren’s syndrome, paeoniflorin reduces ATP-induced pro-inflammatory cytokines IL-1-β and IL-6 secretion by inhibiting P2×7R expression.^76^ Furthermore, paeoniflorin demonstrates activity against COX-1 and COX-2 enzymes, with IC50 values of 11.9 μM and 10.8 μM, respectively.^77^ Collectively, paeoniflorin emerges as a potential drug or lead compound for the prophylaxis and treatment of various inflammatory and/or autoimmune diseases, including rheumatoid arthritis, allergic contact dermatitis, psoriasis, and ulcerative colitis.

### Naringin

Naringin, primarily derived from citrus fruit species, boasts a myriad of beneficial pharmacological features, including anticancer, anti-inflammatory, antioxidant, antiatherogenic, antiulcer, antidiabetic, cardioprotective, and renoprotective properties. With its ability to scavenge free radicals, naringin has garnered significant attention as a natural agent against oxidative stress and kidney injury. Moreover, it demonstrates the capacity to inhibit excessive TNFα release from macrophages in LPS-induced mice models.^78,79^ The existing literature encompasses nearly 4000 studies exploring the effects of naringin in the context of sepsis. One crucial role of naringin is the regulation of inflammation by inhibiting pro-inflammatory signaling pathways in various cell types. For instance, it inhibits TNFα-induced TLR2 expression in differentiated adipocytes by suppressing the activation of NF-κB and c-Jun NH2-terminal kinase pathways.^80^ In hepatocytes, naringin decreases pro-inflammatory cytokines, including TNF-α, interleukin-6, and IL-1-β, by suppressing the NF-κB pathway.^81^ Naringenin also inhibits the production of hyperalgesic cytokines (IL-33, TNF-α, and IL-1-β) and NF-κB activation in paw skin.^82^ Like other phytochemicals, naringin modulates signaling pathways by suppressing the phosphorylation of ERK1/2, JNK, and p38 MAPK.^83^ Most scientific studies have reported the anti-inflammatory activities of naringenin in animal models of inflammation-related diseases, including sepsis and endotoxic shock.^84^ These preclinical studies underscore the therapeutic potential of naringenin in various inflammation-related disease settings with minimal systemic toxicity, warranting further clinical investigation based on the promising preclinical results.

### Baicalein

*Scutellaria baicalensis*, known for its diverse biological effects, including antioxidant, anti-inflammatory, antiapoptotic, and antileukemic activities, features baicalein as its major active component.^78^ The literature currently encompasses nearly 3000 studies exploring the effects of baicalein in the context of sepsis. Baicalein has demonstrated protective effects against endotoxic shock by inhibiting LPS-induced TNF-α and iNOS production. In septic rats, it improves myocardial contractility during LPS-induced sepsis. As an anti-inflammatory agent, baicalein reduces neutrophil migration in the liver, lowers hepatic and circulating proinflammatory cytokine levels, attenuates liver injury, and improves survival following CLP. These findings suggest that baicalein can protect against CLP-induced liver injury by inhibiting the inflammatory response.^85^ Furthermore, baicalein plays a crucial role in preventing cellular damage by inhibiting p38 MAPK and NF-κB signaling and suppressing the increases in JNK and ERK activity.^85^ Despite these promising findings, the specific role of baicalein in polymicrobial sepsis-induced liver injury and its molecular mechanisms remain.

## Conclusion

Sepsis is a leading cause of mortality in critically ill patients, posing a significant economic burden on healthcare systems. The uncontrolled inflammatory response during sepsis development, often leading to multiorgan failure and death, remains a challenge despite advances in antibiotics and supportive care. Factors such as antibiotic resistance, noninfectious causes, and delays in treatment initiation contribute to the complexity of managing sepsis. This review explores alternative treatment strategies focusing on the ameliorative effects of widely consumed phytochemicals and their cellular and molecular mechanisms in various in vitro and in vivo sepsis models. While clinical trials evaluating the effects of phytochemicals for sepsis treatment are lacking, preclinical studies suggest their potential to improve clinical complications. Phytochemicals, known for inhibiting sepsis development, may also mitigate severe sepsis-induced organ injuries, including liver, lung, and renal damage. The discussed phytochemicals act through diverse mechanisms: inhibiting bacterial growth, blocking LPS binding to cell surface receptors, disrupting TLR-4 dimerization and downstream signaling, modulating key signaling molecules and pathways (NF-κB, MAPKs, COX-2, Nrf-2, AP-1, NLRP3 inflammasome, HMGB1, PG-E2), and enhancing antioxidant capacity while reducing oxidative stress. Beyond their ameliorative effects, phytochemicals are considered safe, natural, cost-effective, readily available, and have minimal side effects. Preemptive administration of phytochemicals before sepsis onset appears effective, emphasizing their potential as adjunct or complementary therapeutic agents for critically ill sepsis patients. Despite not being essential nutrients, phytochemicals can be included in treatment regimens to enhance overall effectiveness and expedite the healing process.

## Figures and Tables

**Table 1.** Phytochemicals and Their Beneficial Effects on Different Sepsis Models

Phytochemical	Sepsis Model	Mechanism of Action	Reference
Green tea (EGCG)	CLP-induced sepsis	Improved hypertension Improved survival Inhibited activation of NF-κB Inhibited NOS2 gene expression	^33^
	LPS-induced endotoxemic mice	Inhibited endotoxin-induced release of HMGB1 improved survival Suppression of HMGB1-mediated inflammatory responses	^86^
	LPS-induced inflammation on human peripheral blood mononuclear cells	Reduced the macrophage inflammatory phenotype	^87^
Curcumin	CLP-induced sepsis	Increased T regulatory cells Increased IL-10 Decreased plasma TNFα and IL-6	^88^
	LPS-induced endotoxemic mice	Inhibited signal of NLRP3 inflammasome activation	^89^
	LPS-activated RAW 264.7 cells	Inhibited the production of IL-6 via suppressing the NF-κB signaling and phosphorylation of STAT1	^38^
Resveratrol	Rats with sepsis-induced ALI	Increased HO-1 expression Increased IL-10 level Activated PI3/Nrf2 signaling pathway Decreased MIP-2 level Decreased IL-18 level	^45^
	Sepsis-induced liver injury	Decreased HMGB1 serum level	^46^
	CLP-induced sepsis	Reduced IL-6, IL-1-β, and TNFα Suppressed IRE1-NF-κB pathway Attenuated the overexpressions of GRP78, BiP, phosphorylated IRE1, and p65 proteins Increased IL-10 level	^47^
Quercetin	LPS-induced sepsis	Enhanced macrophage M2 polarization	^90^
	CLP-induced sepsis	Reduced the activities of SOD, CAT, and APX Reduced HMGB1 level Reduced ROS level	^91^
	CLP-induced sepsis	Decreased YKL-40, XO, NO, and MDA Increased antioxidant enzymes levels	^92^
Berberine	CLP-induced sepsis	Decreased TNF-alpha and IL-6 Lower TLR2 and TLR4 expression Upper TLR9 expression	^93^
	CLP-induced sepsis	Activated the Wnt/β-catenin signaling pathway Inhibited claudin-12, β-catenin, VE-cadherin level	^94^
	LPS-induced ARDS rat model	Decreased the production of pro-inflammatory cytokines Inhibited NF-κB signaling pathway activation	^57^
Genistein	LPS-induced endotoxemic rats	Lowered the hypotension caused by endotoxin	^62^
	Mice with LPS-induced DIC RAW 264.7 murine macrophage cells	Improved the morphological structure of the liver and kidney Reduced ALT, AST, and BUN Decreased IL-6, TNFα, IL-1-alpha, and IL-1-β	^63^
	LPS-induced endotoxemic rats	Reduced contractile response Increased nitrite plasma levels Increased iNOS expression	^61^
Apigenin	CLP-induced sepsis	Increased TNF-alpha, IL-1-β, IL-6, and TGF-β levels Reduced IL-10 level Increased CD3 and CD68 positivity Increased NF-κB activation	^65^
	LPS-induced endotoxemic mice	Decreased serum ALT, AST, ALP, γ-GT, CRP, total and direct bilirubin levels, liver MPO activity, MDA, NOx, PGE2, TNF-alpha, IL-1-β, and IL-6 levels, iNOS and COX-2 mRNA levels, phosphorylation of NF-κB, p65, IκB, and IKK proteins Increased serum albumin, total protein, GSH levels, and catalase and SOD activities	^66^
	LPS-induced inflammation on BV2 microglia cells	Induced NF-κB activation Increased GSK3-beta, Nrf2, and HO-1 levels Attenuated TNF-alpha, IL-1-β, and IL-6 productions	^69^
Paeoniflorin	LPS-activated RAW 264.7 cells	Increased the viable cells Decreased NO and PGE2 levels Repair the DNA	^74^
	LPS-induced inflammation on HUVEC cells	Inhibited dextran extravasation Inhibited leukocyte migration through HUVECs	^75^
	CLP-induced sepsis	Decreased IL-1β, TNFα, IL-6, and MDA levels Increased SOD activity Increased Nrf2 and HO-1 expression	^95^
Naringin	LPS-induced endotoxemic mice and RAW 264.7 macrophages	Suppressed TNF-alpha and nitric oxide Improved survival Suppressed expression of iNOS, TNFα inducible COX-2, and IL-6 Inhibited NF-κB expression	^96^
	CLP-induced sepsis	Decreased inflammatory cytokine levels Increased MDA and SOD activities Decreased glutathione level	^79^
	CLP-induced sepsis	Increase in the positivities of CD3, CD68, and NF-κB markers Suppressed pro-inflammatory cytokine levels Increased anti-inflammatory cytokine levels	^78^
Baicalein	LPS-induced sepsis	Decreased the level of iNOS Improved survival Reduced the infiltration of neutrophils into the liver and lungs of rats	^97^
	CLP-induced sepsis	Increased CD3, CD68, and NF-κB positivity Suppressed pro-inflammatory cytokine levels Increased anti-inflammatory cytokine levels	^78^
	LPS-induced RAW 264.7 cells	Inhibited the release of TNFα Decreased IL-6, iNOS, and NO levels	^9^

ALP, alkaline phosphatase; ALT, alanine transaminase; AST, aspartate transaminase; BUN, blood urea nitrogen; CRP, C-reactive protein; γ-GT, gamma-glutamyl transferase; GSH, glutathione; HUVECs, human umbilical vein endothelial cells; IL, interleukin; iNOS, inducible nitric oxide synthase; MDA, malondialdehyde; MPO, myeloperoxidase; NF-κB, nuclear factor kappa B; SOD, superoxide dismutase; TNFα, tumor necrosis factor alpha.
